# Towards a Microwave Imaging System for Continuous Monitoring of Liver Tumor Ablation: Design and In Silico Validation of an Experimental Setup

**DOI:** 10.3390/diagnostics11050866

**Published:** 2021-05-11

**Authors:** Mengchu Wang, Rosa Scapaticci, Marta Cavagnaro, Lorenzo Crocco

**Affiliations:** 1 Institute for the Electromagnetic Sensing of the Environment, National Research Council of Italy, 80124 Napoli, Italy; scapaticci.r@irea.cnr.it (R.S.); marta.cavagnaro@uniroma1.it (M.C.); crocco.l@irea.cnr.it (L.C.); 2 Department of Information Engineering, Electronics and Telecommunications, Sapienza University of Rome, 00184 Rome, Italy

**Keywords:** microwave imaging, liver tumor ablation, imaging-guided, dielectric properties, medical imaging system

## Abstract

Liver cancer is one of the most common liver malignancies worldwide. Thermal ablation has been recognized as a promising method for its treatment, with a significant impact on clinical practice. However, the treatment’s effectiveness is heavily dependent on the experience of the clinician and would improve if paired with an image-guidance device for treatment monitoring. Conventional imaging modalities, such as computed tomography, ultrasound, and magnetic resonance imaging, show some disadvantages, motivating interest in alternative technologies. In this framework, microwave imaging was recently proposed as a potential candidate, being capable of implementing real-time monitoring by means of low-cost and portable devices. In this work, the in silico assessment of a microwave imaging device specifically designed for liver ablation monitoring is presented. To this end, an imaging experiment involving eight Vivaldi antennas in an array configuration and a practically realizable liver phantom mimicking the evolving treatment was simulated. In particular, since the actual phantom will be realized by 3D printing technology, the effect of the plastic shells containing tissues mimicking materials was investigated and discussed. The outcomes of this study confirm that the presence of printing materials does not impair the significance of the experiments and that the designed device is capable of providing 3D images of the ablated region conveying information on its extent and evolution. Moreover, the observed results suggest possible improvements to the system, paving the way for the next stage in which the device will be implemented and experimentally assessed in the same conditions as those simulated in this study.

## 1. Introduction

Liver cancer is considered a major health problem worldwide. Indeed, it is among the most common liver malignancies [1], being ranked fifth in men and eighth in women among all cancer diseases [2]. However, despite advanced diagnosis and treatment methods, liver cancer still presents an increasing yearly death rate [3]. The majority of liver cancers is attributed to the infection of chronic hepatitis B (HBV)/hepatitis C virus (HCV) and alcohol abuse [4]. Over 80% of liver cancers occur in developing countries due to the lack of infrastructure and poor management of the disease [4].

Over the last decades, thermal ablation therapies have been intensively investigated for the treatment of liver tumors as an alternative to standard surgical approaches [5]. Thermal ablation aims at destroying the malignant cells by dramatically increasing or decreasing the temperature in the tissue [5]. It can treat liver tumors with a minimally invasive approach, without damaging adjacent vital structures [6] and with a significant reduction in morbidity and mortality as compared to other treatment procedures [7]. Moreover, thermal ablation, and in particular modalities based on electromagnetic power deposition, such as RF and microwave ablation (MWA), show attractive features such as low-cost, rapidity of the procedure, the use of applicators with very small dimensions, and the applicability to patients not suited for surgical procedures. However, through the years, the diversity of outcomes has led to a bottleneck in the development of ablation treatments [8], which mostly rely on the clinician’s expertise and skills. In this respect, the lack of an accurate and objective real-time imaging system to be operated during the treatment for monitoring purposes is one of the most important shortcomings of the actual systems, and, as a consequence, a fundamental task that researchers must face to allow the deployment of thermal ablation to reach its full potential [9].

To date, several conventional imaging modalities, such as magnetic resonance imaging (MRI), computed tomography (CT), ultrasound (US), fluoroscopy, and positron emission tomography (PET), have been experimented with for ablation treatment monitoring, in some cases in clinical settings [9]. The mentioned imaging modalities play an important role for different purposes along the thermal procedure: (1) *treatment planning*, determining the tumor size, shape, and location relative to the blood vessels, etc.; (2) *treatment targeting*, defining the placement of the applicator before the treatment procedure and the resolution of the best route to achieve such a placement; (3) *treatment assessment*, assessing after the ablation procedure if the target endpoint was reached [9]. However, the *continuous monitoring* of the evolution of the ablation zone during the treatment remains difficult. MRI currently shows well-validated techniques for near real-time temperature monitoring [9], but its high cost, bulky size, and poor compatibility with the applicators make its use as a tool to monitor the ablation process on the clinical side unfavorable. CT also has the potential to provide real-time imaging during the treatment. However, the ionizing radiation it exploits has an intrinsic cancer risk, both for the patient and for the medical staff [10]. Finally, US has attractive features such as cost-effectiveness and real-time imaging capability. However, it is difficult to monitor the ablation process because the US sensor is blinded by a hyper-echogenic cloud caused by water vaporization [9,11].

Microwave imaging (MWI) is a modality that has been proposed for medical diagnostics in recent years and the implementation of MWI medical devices has been intensively investigated by researchers over the world in the last two decades [12,13,14,15,16]. As compared to the conventional imaging modalities mentioned above, MWI shows attractive characteristics for thermal ablation monitoring, being non-ionizing, of compact size, and cost effective [17]. Moreover, MWI has the potential to provide real-time monitoring of the ablation zone during the treatment [12].

MWI images the variations in the electromagnetic properties with respect to an unperturbed situation by recording (and properly processing) the electromagnetic field backscattered by the region of interest when probed by a known incident wave. Since tissue dielectric properties change during the thermal ablation procedure as a function of temperature [18], the ablation process creates a contrast between the dielectric properties in the pre-ablation and post-ablation stages. By taking advantage of this contrast, which evolves during the treatment, it is possible to retrieve the changes in the permittivity of the ablated region and, hence, the temperature. For this reason, efforts have been addressed to develop MWI devices for real-time monitoring of thermal therapies in different anatomical regions such as liver [19,20], breast [21], and brain [22]. However, MWI shows drawbacks in terms of resolution and penetration depth. To this end, accurate experimental validations in controlled and reproducible conditions are needed to assess its actual feasibility.

In view of the above, this paper presents the in silico assessment of the experimental setup for monitoring liver ablation described in [19], with the aim of evaluating its anticipated performances before realization. In addition to the abovementioned reasons, the importance of such a preliminary numerical validation stands in the fact that the realization of the non-homogeneous phantoms used in the controlled experiments necessarily require the use of materials (such as plastic) that have dielectric properties which are significantly different from those of human body tissue in which MWA is usually performed, e.g., the liver. Accordingly, the presence of such materials could affect the evaluation of the performances of the MWI system in a non-realistic way. Numerical simulations were herein carried out to understand the effect of the plastic shells containing tissue-mimicking material by comparing the imaging results in the presence and absence of them.

As a main outcome, this paper proves that the presence of plastic shells does not affect the meaningfulness of the experiments as well as imaging results, so that the designed controlled experiment is a valid means to assess the feasibility of an MWI device for monitoring liver ablation. In this respect, the 3D images obtained in a setting simulating the working conditions of the considered device show that, even in the presence of the unavoidable measurement noise on data and without the aid of pre-operatory a priori information (e.g., an image of the scenario under testing obtained with another imaging modality), it is possible to localize the changes occurring within the ablated region and appraise its extent in real time. Finally, the results of the in silico experiments also allow to foresee in which directions the system can be improved, both in terms of hardware and processing of the data.

## 2. Materials and Methods

### 2.1. Description of the MWI Device

The main design guidelines for the microwave imaging system for liver ablation monitoring herein studied (i.e., the working frequency, the coupling medium, and the antennas to be adopted) are given in [19]. In particular, it was shown that the 0.5–2 GHz frequency band and a coupling medium with properties equal to ε_CM_ = 23 and σ_CM_ = 0.07 S/m are a good trade-off between penetration depth, imaging resolution, and antenna dimension. As far as the antenna is concerned, a suitable candidate was identified as a Vivaldi antenna, having a dimension of 60 × 60 mm^2^ and working frequency from 0.5 to 5 GHz, with the possibility to work even at higher frequencies [19]. Such an antenna radiates a linearly polarized field and has an end-fire radiation pattern, so that several radiating elements can be allocated in an array configuration. In particular, the MWI device studied in this paper exploits an array of 8 antennas, which represents a good trade-off among system complexity, antenna mutual coupling, and imaging capabilities. The distance between each antenna was 23 mm, which corresponded to a distance larger than a quarter of the wavelength (λ/4) at 1 GHz in the coupling medium. In the device, all antennas worked both as transmitters and receivers in order to implement a multi-static measurement configuration in which when one of the antennas is excited all the others record the signals scattered by the observed region.

### 2.2. Outline of the Setup

**Figure 1:**
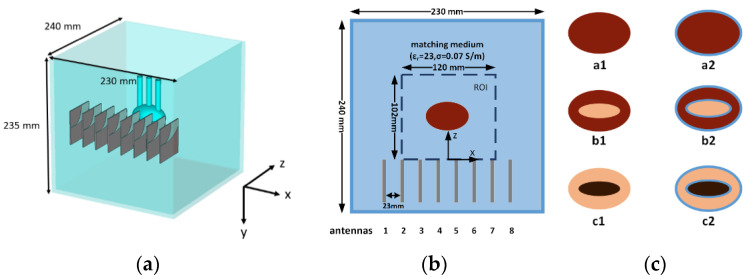
(**a**) The experimental setup; (**b**) top view of the setup configuration; (**c**) the different phantoms considered in the numerical study.

The experimental setup is shown in Figure 1a, and its top view (the projection in the x-z plane) is shown in Figure 1b. The setup consists of a container made of acrylonitrile butadiene styrene (ABS) having a size of 230 × 240 × 235 mm^3^, (width × length × height). The container is filled with the coupling medium identified in [19]. Such a medium was designed in such a way to maximize the power delivered to the liver, so that, from an electromagnetic point of view, it represents an effective medium model of the whole abdomen region (and its different tissues) [19]. The antenna array was inserted into the container, completely immersed into the coupling medium, and placed close to one side of the container. Finally, still completely immersed in the coupling medium, a phantom could be located 35 mm away from the antennas array as detailed in the following paragraph. In Figure 1b, the region of interest (ROI) for the imaging system, which was 120 mm × 102 mm × 102 mm wide, was marked.

### 2.3. Phantom Mimicking the Ablated Region

As far as the development of the phantom was concerned, a simple ellipsoidal structure was designed to mimic the thermally ablated area evolution during the ablation treatment. Such a phantom is sufficiently accurate for the study, as the ablation zone typically shows an ellipsoidal shape [23]. The dimensions of the phantom were translated from the experimental results in [23], while the dielectric properties of the pre-ablation liver and the coagulation necrosis region were adopted from frequency-dependent measured data [18,24]. The dielectric properties of the carbonized liver were from [18], which were obtained at a single frequency (ε_r_ = 8.33, σ = 0.39 S/m, @2.45 GHz). In more detail, the phantom consisted of two nested ellipsoids, the outer one having axes with dimensions of 60 mm × 40 mm × 40 mm (x−y−z) and the inner one with dimensions of 34 mm × 13 mm × 13 mm (x−y−z). The origin of the *z*-axis is positioned at the tip of the antennas.

By filling the two ellipsoidal regions with different tissue-mimicking materials, it was possible to simulate different stages of the treatment, namely (Figure 1c):

(a) pre-treatment scenario: both ellipsoids are filled with the liver-mimicking material (phantom a1 Figure 1c);
(b) ongoing ablation scenario: the outer ellipsoid is filled with the liver mimicking material and the inner one with coagulation necrosis-mimicking material (phantom b1 Figure 1c);
(c) completed treatment scenario: the outer ellipsoid is filled with the coagulation necrosis mimicking material and the inner one is filled with the carbonized-tissue-mimicking material (phantom c1 Figure 1c).

Since the actual phantom will be fabricated with 3D-printed 1.5 mm thick ABS structures (ε_r_ = 3, σ = 4 × 10^−3^ S/m), it was necessary to understand the possible perturbations of the underlying electromagnetic phenomena that could possibly arise due to the presence of the ABS shells. To this end, two sets of numerical phantoms were considered in the study, one without the ABS shells—as described above—and one with the ABS shells. In particular, as shown in Figure 1, phantoms labeled as (a1), (b1), and (c1) refer to ABS-free phantoms (e.g., representing an actual ex-vivo experiment), whereas phantoms (a2), (b2), and (c2) include the ABS shells.

Finally, we denote as s0, the initial or reference scenario, i.e., when no phantom is present in the container.

### 2.4. Electromagnetic Simulations

The different experiments, involving the described device, and the different phantom configurations, were simulated using the CST software (Dassault Systèmes, Vélizy-Villacoublay, France). CST is an electromagnetic simulation software based on the finite integration technique applied to Maxwell’s curl equation in the time domain [25].

The different scenarios were simulated and the electromagnetic field into the studied domain was evaluated. In particular, as concerns the relevant MWI quantities, five different frequencies were considered: 600 MHz, 800 MHz, 1000 MHz, 1200 MHz, and 1400 MHz, chosen in agreement with the guidelines in [19]. For each frequency *f* and each scenario, the simulation provides the incident field E_i_(r,*f*) = (E_ix_(r,*f*), E_iy_(r,*f*), E_iz_(r,*f*)) inside the ROI, i.e., the field radiated by each transmitter when no phantom was present (initial state, s0) as well as the 8 × 8 scattering matrix **S** encoding the scattering parameters of the antennas in the MWI array. In particular, the elements on the diagonal of **S** were the eight reflection parameters (one for each antenna in the array, *S_ii_*) while the off-diagonal elements were the 56 transmission parameters (accounting for all possible antenna combinations in the array, *S_ij_*).

To run the simulation, the ROI was discretized into cubic voxels of 3 mm sides. The total voxel number was 50,225 (41 × 35 × 35). Moreover, to simulate measurement noise, the simulated scattering parameters were corrupted with an additive Gaussian noise with SNR = 45 dB, which is fully consistent with the performance of commercial vector network analyzers (VNAs) in terms of noise floor and dynamic range.

### 2.5. Image Formation Algorithm

The simulated fields and scattering parameters provide the quantities needed to mimic the imaging experiments with the proposed setup.

The imaging step was carried out by means of a differential MWI approach similar to the one used in [12], in agreement with the fact that the evolution of the treatment was the goal of the imaging task. In such an approach, the imaging task was faced by exploiting the distorted Born approximation (DBA) [17], i.e., assuming that the total field actually induced by each antenna in the ROI, say *E_t_*, can be approximated with the incident field, ${\underline{E}}_{t}≅{\underline{E}}_{i}$. By so doing, the inverse scattering problem underlying the imaging task is reduced to a linear ill-posed inverse problem [26]. Such a simplification has the remarkable consequence of enabling real-time imaging results, which is a key requirement to perform treatment monitoring. On the other hand, this approach can only provide information on the morphology (i.e., presence, position, extent) of the region wherein the contrast variation is occurring, since the quantitative estimation of the tissue properties is only achieved when the enforced approximation is completely fulfilled. While a perspective goal is, of course, that of obtaining an estimate of the permittivity in the treated tissue, using for instance the iterated (non-real-time) version of the DBA as in [22], the peculiarities of the observed scenario (in which changes are necessarily related to the evolving treatment), make the appraisal of the position and size of the variation a clinically relevant information.

The input datum of the imaging procedure was given by the differential scattering parameters calculated as the difference between the (noise corrupted) scattering parameters simulated in two different scenarios. From all the simulated scenarios, ten differential data sets were generated, namely: aX-s0, bX-s0, cX-s0, bX-aX, cX-bX, where X is equal to 1 or 2 according to the considered phantom (see Figure 1). The first data sets accounted for the changes that occurred with respect to the reference scenario, and therefore the goal was to image the extent of the outer ellipsoid. Conversely, the bX-aX data set was representative of a situation in which the changes occur only in the inner ellipsoid, wherein the tissue ablated in the initial part of the treatment was located. Finally, cX-bX accounted for the difference between the intermediate and final stages of the treatment, and the goal was to image the external ellipsoid and possibly the transition from necrosis to carbonization occurring in the inner part. It is to be noted here that an imaging of the a1-a2 or b1-b2 was not within the scope of this work, because it would give an image of the shells, while the aim of this study was to appraise to what extent the unavoidable presence of the shells can deviate the experiment’s results with phantom from the ones achieved in the real situation. For the generic data set, let us denote with **∆S** the 320 element vector containing the complex values of the differential scattering parameters (that is considering the available 5 frequencies and the 64 scattering parameters), obtained by reshaping into a vector the five 8 × 8 differential scattering matrices.

The kernel of the imaging algorithm is given by the 320 × 50,225 matrix **K**, which has a generic element, *K_mn_*, given by:

(1) $$K_{mn}=\frac{-j}{4}\omega_{f}\varepsilon_{eqCM}{\underline{E}}_{i}\left(r_{n},p\right)\cdot {\underline{E}}_{i}\left(r_{n},q\right)$$

where *n* = 1,…, 50,225 denotes the *n*-th voxel of the ROI, $\omega_{f}$ is the angular frequency, with *f* = 1,.., 5 denoting the relevant frequency, $\varepsilon_{eqCM}=\varepsilon_{r}\varepsilon_{0}+j\frac{\sigma }{\omega }$ is the complex equivalent permittivity of the coupling medium, *p* = 1,.., 8 denotes the transmitting antenna in the array and *q* = 1,.., 8 the receiving antenna. The *f*, *p,* and *q* indices are organized in the same way as the data vector **∆S**.

Let **∆x** denote the 50,225 × 1 vector, which has elements that are the values of the unknown contrast in the voxels of the ROI, i.e., the variation of the dielectric properties within the ROI for the different data sets.

With respect to the above, the matrix equation to be solved reads:

(2) ΔS=KΔx

Since the kernel matrix **K** is ill-conditioned, Equation (2) cannot be solved via direct matrix inversion, but must be solved in a generalized sense, introducing some form of regularization. To this end, let us define the singular value decomposition (SVD) of **K** as [27]:

(3) $$K=U\cdot \sum \cdot V^{H}$$

where **U** is the matrix of the left singular vectors of **K**, **V** is the matrix of the right singular vectors of **K**, **∑** is a diagonal matrix of the singular values, and ${}^{H}$ stands for the conjugate transpose. The diagonal of **∑** is populated with the singular values $\sigma_{i}$ of **K**, which are real scalars, approaching zero as: $\sigma_{1}\geq \sigma_{2}\geq \ldots \geq 0$. Once **K** is evaluated, the problem is solved in a regularized sense using the truncated singular value decomposition (TSVD) algorithm [12] that provides an explicit inversion formula for Equation (2) that reads:

(4) $$\Delta x=V\Sigma_{\nu }^{-1}U^{H}\Delta S$$

where ${}^{-1}$ denotes the inverse, and $\Sigma_{\nu }^{}$ is the same matrix as Σ except that it contains only the singular values that have an index which is lower than the threshold index ν (the other singular values are replaced by zeroes). The threshold ν acts as a regularization parameter. Such a regularization parameter is chosen as a trade-off between the accuracy and the stability of the approximation, depending on the noise level. In particular, larger values of ν lead to higher accuracy but lower stability of the estimated solution [27]. Typically, the candidate values of ν correspond to the indices where the singular value curve presents changes in slope.

As the imaging algorithm provides qualitative information only, the estimated Δx is turned into its normalized absolute value, I, which represents the final output of the imaging procedure.

The SVD also provides a way to appraise the achieved results with respect to the best possible result that can be obtained by means of the adopted reconstruction algorithm. Such an ideal reconstruction, say $\Delta x_{ID}$, is computed for each scenario as:

(5) $$\Delta x_{ID}=V_{\nu }^{}V_{\nu }^{H}\;\Delta x_{GT}$$

where $\Delta x_{GT}$ is the ground truth (i.e., the contrast used to generate the data), and $V_{\nu }^{}$ is a N×ν matrix whose columns are the first ν right singular vectors of K. The quality of the reconstruction is finally appraised by means of the normalized mean square error (NMSE) defined as:

(6) $$\mathrm{NMSE}=\underset{voxels}{\sum }\frac{{\left|I_{ID}-I\right|}^{2}}{{\left|I_{ID}\right|}^{2}}$$

where $I_{ID}$ denotes the normalized absolute value of $\Delta x_{ID}$.

## 3. Results

### 3.1. Signal Level

The first analysis carried out in this paper was concerned with the comparison of the signal level associated to the scattering matrices simulated in the different scenarios. To this end, the L2 norm of each matrix, i.e.,

(7) $$S=\sqrt{\sum_{i,j=1}^{8}{\left|S_{ij}\right|}^{2}}$$

was computed. The obtained values are reported in Table 1.

Then, the L2 norm of the differential scattering matrices ΔS was computed. In this case, the norm was normalized to one of the s0 data sets and expressed in dB to appraise the useful signal level with respect to the one measured in the reference condition (i.e., the one with respect to which the imaging kernel matrix **K** was built). The values of the norm are reported in Table 2 and Table 3.

### 3.2. Imaging Results

**Figure 2:**
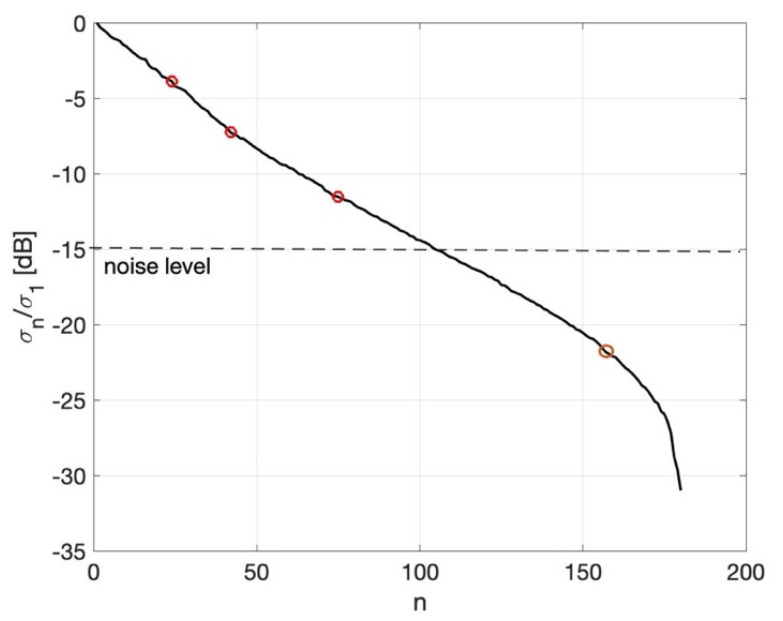
Plot of the normalized singular values in dB. The red circles denote the candidate threshold indices. The noise level (with respect to the differential signal) is shown as a dashed line.

In Figure 2, the plot of the normalized singular values $\sigma_{n}$ in the dB scale is reported. As can be appreciated, the curve exhibits four changes of slope at *n* = 24, 45, 75, and 155, and these points are marked on the curve. As discussed in the next section, the threshold index ν = 75 was used in the TSVD inversion Equation (4).

**Figure 3:**
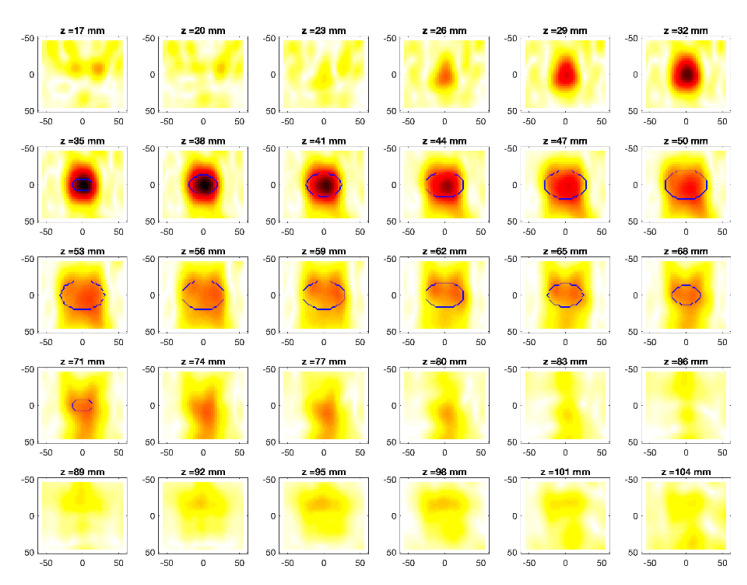
MWI reconstructions for the a1-s0 data set as slices in the *x-y* plane for various values of *z*. In the panels, the *x*-axis is the horizontal axis, and the *y*-axis is the vertical one (see Figure 1 for the reference system adopted). Units are in mm. Reconstructed contrast in normalized amplitude, color bar ranges from 0 (white) to 1 (dark red).

**Figure 4:**
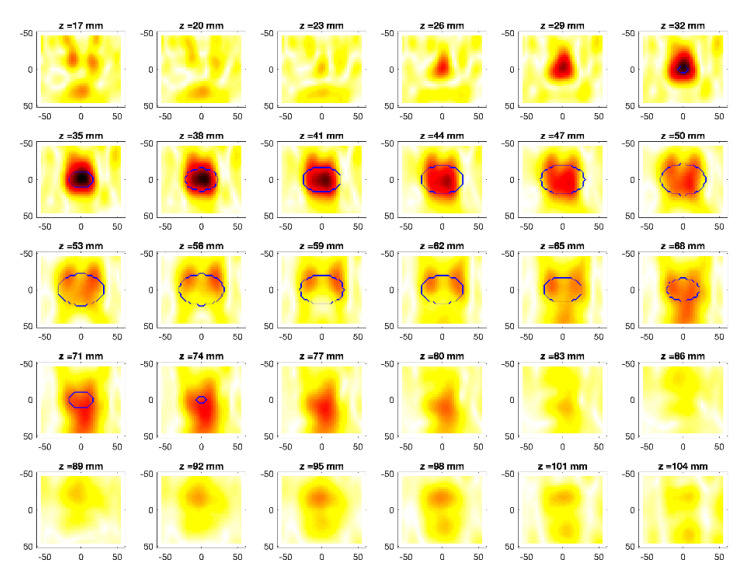
MWI reconstructions for the a2-s0 data set as slices in the *x-y* plane for various values of *z*. In the panels, the *x*-axis is the horizontal axis, and the *y*-axis is the vertical one (see Figure 1 for the reference system adopted). Units are in mm. Reconstructed contrast in normalized amplitude, color bar ranges from 0 (white) to 1 (dark red).

**Figure 5:**
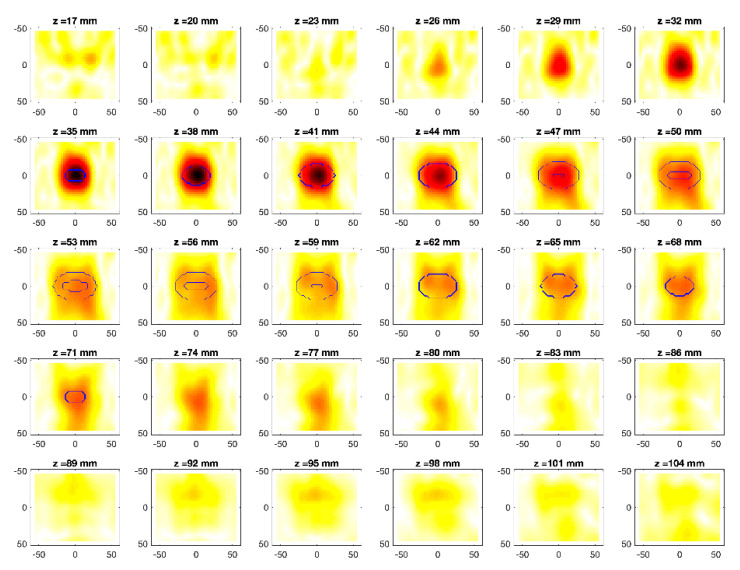
MWI reconstructions for the b1-s0 data set as slices in the *x-y* plane for various values of *z*. In the panels, the *x*-axis is the horizontal axis, and the *y*-axis is the vertical one (see Figure 1 for the reference system adopted). Units are in mm. Reconstructed contrast in normalized amplitude, color bar ranges from 0 (white) to 1 (dark red).

**Figure 6:**
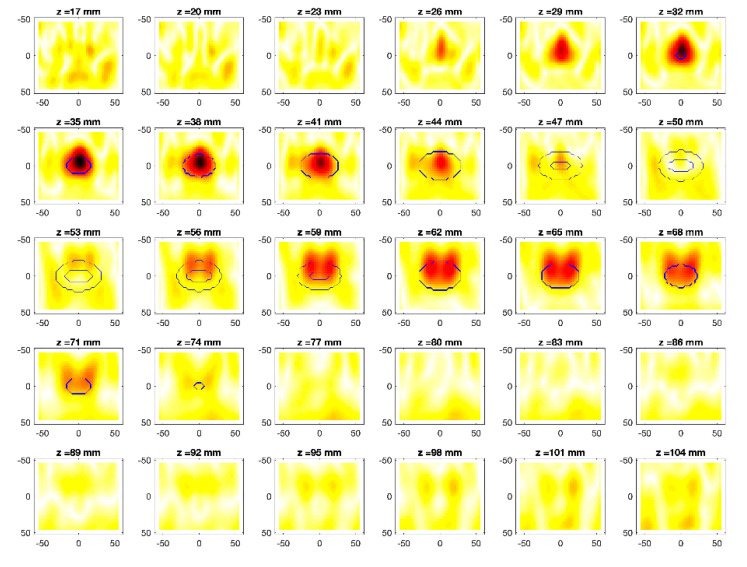
MWI reconstructions for the b2-s0 data set as slices in the *x-y* plane for various values of *z*. In the panels, the *x*-axis is the horizontal axis, and the *y*-axis is the vertical one (see Figure 1 for the reference system adopted). Units are in mm. Reconstructed contrast in normalized amplitude, color bar ranges from 0 (white) to 1 (dark red).

**Figure 7:**
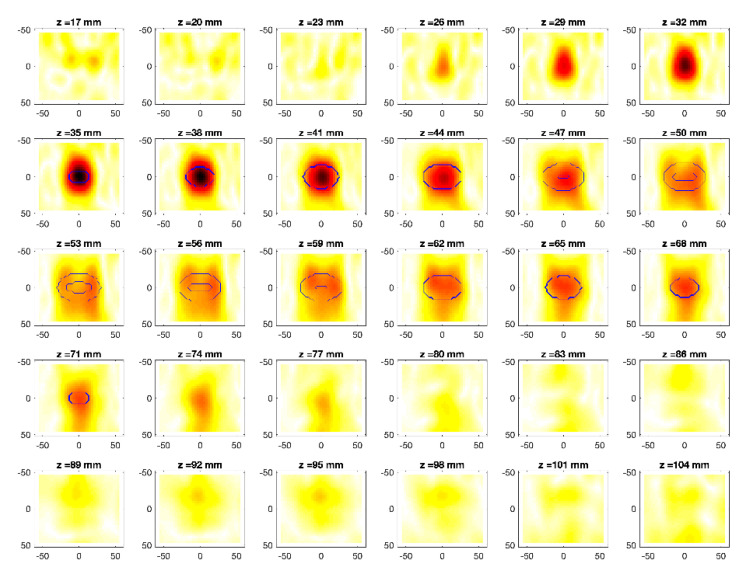
MWI reconstructions for the c1-s0 as slices in the *x-y* plane for various values of *z*. In the panels, the *x*-axis is the horizontal axis, and the *y*-axis is the vertical one (see Figure 1 for the reference system adopted). Units are in mm. Reconstructed contrast in normalized amplitude, color bar ranges from 0 (white) to 1 (dark red).

**Figure 8:**
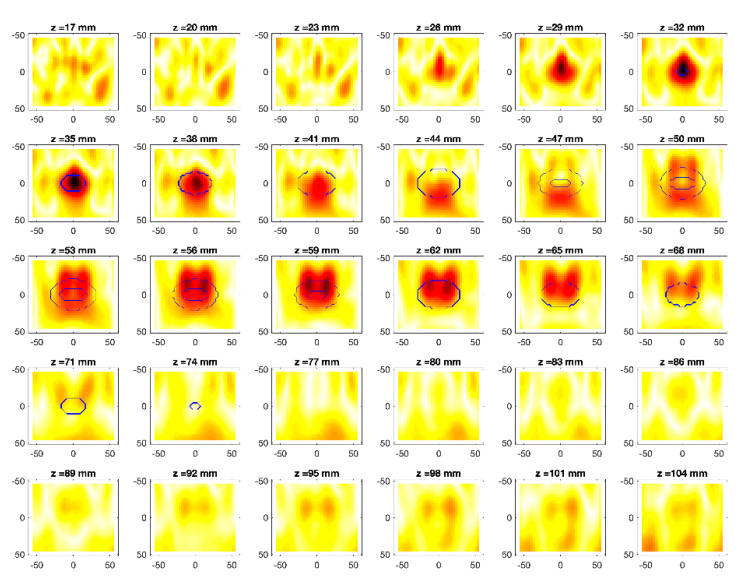
MWI reconstructions for the c2-s0 data set as slices in the *x-y* plane for various values of z. In the panels, the *x*-axis is the horizontal axis, and the *y*-axis is the vertical one (see Figure 1 for the reference system adopted). Units are in mm. Reconstructed contrast in normalized amplitude, color bar ranges from 0 (white) to 1 (dark red).

**Figure 9:**
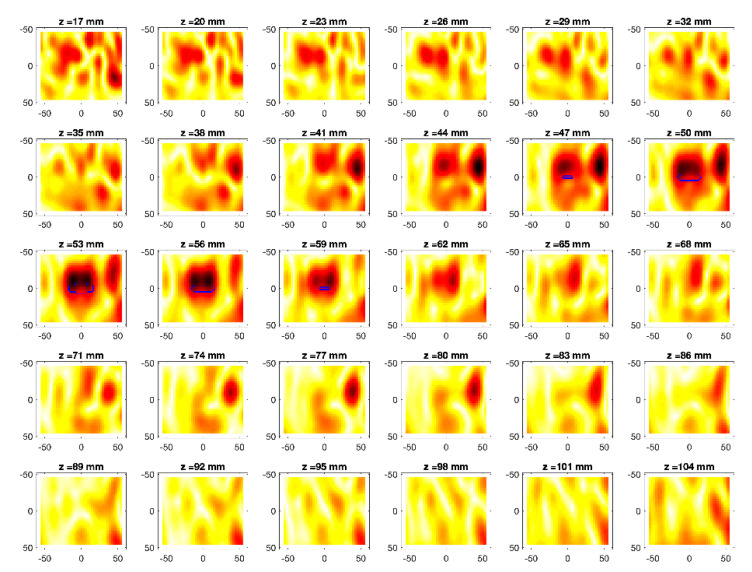
MWI reconstructions for the b1-a1 data set as slices in the *x-y* plane for various values of z. Note that in this case, only the inner ellipsoid of the actual contour is shown, as this experiment aimed only at imaging variations occurring in the core region of the phantom. In the panels, the *x*-axis is the horizontal axis, and the *y*-axis is the vertical one (see Figure 1). Units are in mm. Reconstructed contrast in normalized amplitude, color bar ranges from from 0 (white) to 1 (dark red).

The obtained MWI reconstructions are shown in Figure 3, Figure 4, Figure 5, Figure 6, Figure 7, Figure 8, Figure 9, Figrue 10, Figure 11 and Figure 12 for all the considered scenarios; in the figures, the cross-sections along the *z*-axis (i.e., moving from the antenna towards the phantom) are shown. To improve readability, the contour of the phantom was superimposed on the reconstruction in the relevant cross-sections. For the sake of the readability of the results, the visualization of the imaging outcomes was limited to the range x = −60–60 mm, y = −51–51 mm, and z = 17–104 mm. In particular, the voxel layers on the boundary of the ROI close to the antennas were not shown to avoid artifacts which were due to the antennas’ presence.

To appraise the quality of the results, the NMSE with respect to the ideal reconstruction was computed for all cases. The NMSE values are reported in Table 4.

## 4. Discussion

The analysis of the norm of the scattering matrices (Table 1) shows that the signal level in all experiments was the same, regardless of the presence of the ABS shells. Additionally, it is interesting to note that in all cases, the highest frequency gave the lowest signal level, linked to the reduced penetration depth and, as a consequence, sensibility of the imaging approach. As a consequence, both situations (i.e., presence or absence of ABS shells) can be considered equivalent as far as the expected signal level is concerned. On the other hand, the reference scenario s0 gives a comparable signal level as well, so that it follows that the measured signal is dominated by the antenna crosstalk, the useful information being embedded in the differential data (as expected).

For such a reason, it is crucial to analyze the norms of the differential scattering matrices (Table 2 and Table 3). These latter are computed with respect to the reference scenario to provide a homogeneous evaluation. Comparing the cases with and without ABS shells, it was found that the signal level was almost always comparable (but for the b–a pair), confirming that the designed experiment was reliable, since the dynamic range of the measurement system needed to appraise the useful differential signal was the same as it would be in the “real” case, i.e., when no shells were present.

To build the images, the TSVD threshold index had to be appropriately selected, as discussed in Section 2. In particular, among the possible candidates (*n* = 24, 45, 75, and 155), the TSVD threshold index was selected according to the following observation. The noise level on the measured data was −45 dB, whereas the energy of the differential signals was, on average, approximately −30 dB (see Table 2 and Table 3). Hence, we can conclude that the noise level on the differential signal was, on average, −15 dB. Accordingly, in order to mitigate the impact of the noise on the results and improve their accuracy (which means retaining as many singular values as possible in the TSVD formula), the threshold index was set at ν = 75, as this corresponded to the point at which the change in the slope of the singular value curve occurred just above the level of the noise expected on the differential signal (see Figure 2). Whereas, selecting lower truncation indices (e.g., 24 and 42) would entail a loss of a significant part of the available information, selecting the highest threshold (155) would lead to results dominated by the presence of noise and, hence, be unstable. Note that even if the considered noise level was reasonable if compared to the noise floor of a typical VNA, the above observations also suggest that a slightly larger noise level can be tolerated.

The analysis of the NMSE (Table 4) provides a quantitative assessment of the results obtained applying the TSVD algorithm to the different data sets and shows that they are quite close to the corresponding ideal reconstructions. The larger NMSE value observed for the case with ABS shells has to be attributed to the increased difficulty of retrieving the steep contrast variation due to the ABS shells.

**Figure 10:**
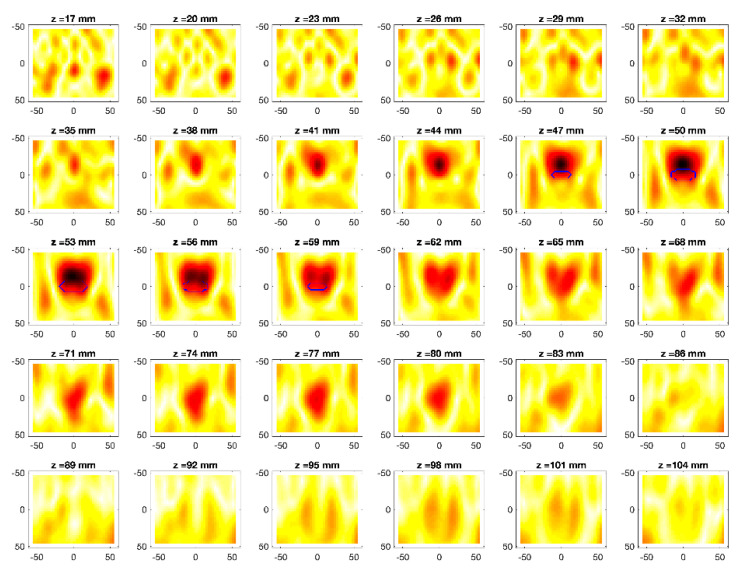
MWI reconstructions for the b2-a2 data set as slices in the *x-y* plane for various values of *z*. Note in this case, only the inner ellipsoid of the actual contour is shown, as this experiment aimed only at imaging variations occurring in the core region of the phantom data set. In the panels, the *x*-axis is the horizontal axis, and the *y*-axis is the vertical one (see Figure 1). Units are in mm. Reconstructed contrast in normalized amplitude, color bar ranges from 0 (white) to 1 (dark red).

**Figure 11:**
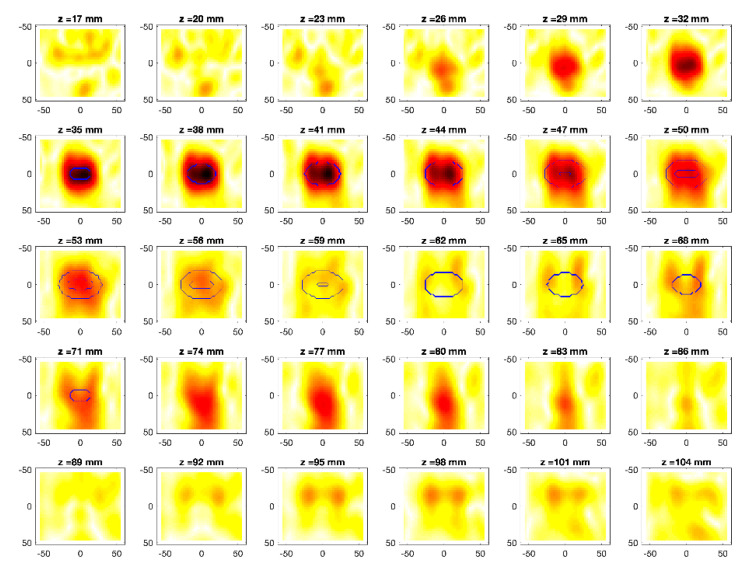
MWI reconstructions for the c1-b1 data set as slices in the *x-y* plane for various values of z. In the panels, the *x*-axis is the horizontal axis, and the *y*-axis is the vertical one (see Figure 1 for the reference system adopted). Units are in mm. Reconstructed contrast in normalized amplitude, color bar ranges from 0 (white) to 1 (dark red).

**Figure 12:**
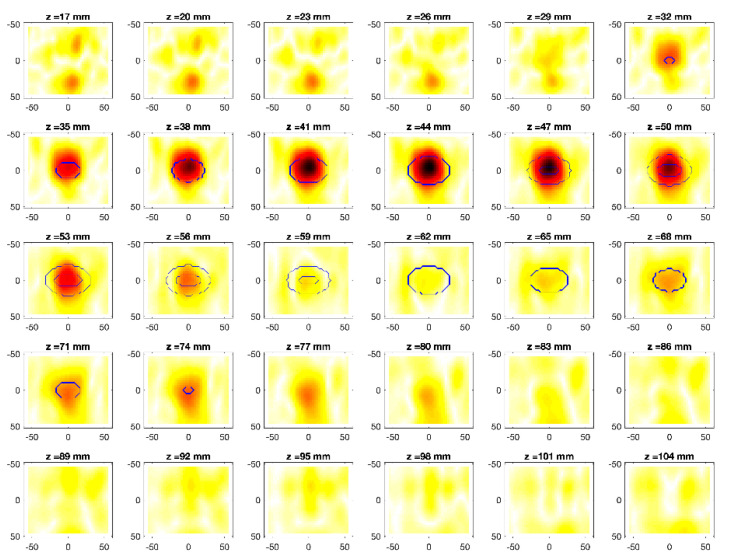
MWI reconstructions for the c2-b2 data set as slices in the *x-y* plane for various values of *z*. In the panels, the *x*-axis is the horizontal axis, and the *y*-axis is the vertical one (see Figure 1 for the reference system adopted). Units are in mm. Reconstructed contrast in normalized amplitude, color bar ranges from 0 (white) to 1 (dark red).

From a qualitative point of view, the imaging results in Figure 3, Figure 4, Figure 5, Figure 6, Figure 7, Figure 8, Figure 9, Figrue 10, Figure 11 and Figure 12 show that regardless of the presence of the ABS shells, the two classes of phantoms lead to comparable results, supporting the validity of the designed experimental setup. In particular:

- In the aX-s0, bX-s0, and cX-s0 cases (Figure 3, Figure 4, Figure 5, Figure 6, Figure 7 and Figure 8), wherein the goal is to image the external ellipsoid, the target always appeared in correspondence of the same section, i.e., for the same values of *z* (*z* = 26 mm), that is 6 mm before the actual position, regardless of the presence of the ABS shell.
- For the bX-aX case (Figure 9 and Figure 10), in agreement with the lower signal level, the images were less clear, especially for the phantom without ABS. However, for the phantom with the ABS shells (where the signal level was indeed slightly higher), it was possible to image the region where the ablation was occurring (i.e., the inner ellipsoid). The target appeared at *z* = 38 mm, i.e., 9 mm before the actual position. The higher signal level and, consequently, better reconstruction achieved when the ABS shell was present can be explained with the higher dielectric contrast introduced by the shell with respect the tissue-mimicking materials. It is worth noting that in the bX-aX case, there was an additional difficulty due to the fact that the DBA was far from being fulfilled. As a matter of fact, for a proper formulation of the DBA in this case, the total field in the scenario a should be considered. However, such a field cannot be faithfully estimated (different from the one at s0) and, therefore, the incident field was used for the data processing, even if it represents a less appropriate choice.
- Finally, for the cX-bX case (wherein a proper formulation of the DBA would require using the field in the b state as well, but the signal level was higher (Figure 11 and Figure 12)), the images obtained with the ABS-free phantom present the same overestimation as the case with the reference c1-s0 case, whereas slightly better results were obtained with the phantom including the ABS shells. However, as these latter could be responsible for this outcome, it can be concluded that in the worst case, the same overestimation as for the aX-s0, bX-s0, and cX-s0 cases has to be expected.

Overall, in all cases the features of the evolving scenarios were quite clearly identified in terms of the maximum extent of the region where the variation was occurring, despite the unavoidable limitations coming from the aspect-limited measurement configuration (i.e., the antennas look at the target only from one side), the limited aperture (only eight antennas are used), and the limited resolution along the *y*-axis (the antennas were only along the *x*–*z* plane). In particular, this latter limitation resulted in an obvious degradation of the estimated volume along the *y*-axis, in agreement with the fact that a 3D image is being reconstructed from data measured by a linear array. Moreover, the occurrence of artifacts, which is due to the presence of noise, does not impair the interpretation of the results (but for the b1-a1 case). Finally, it is worth remarking that all the 3D images were obtained in real time. As a matter of fact, the only computationally intensive part of the algorithm was the evaluation of the SVD of ***K***, which can be done off-line, being independent of the scenario.

As the goal of the performed numerical study was to investigate the feasibility of an experimental setup to prove the capability of the MWI to monitor the real-time MWA treatment of liver tumors, the obtained results confirm that the designed setup can fulfill this purpose, despite the presence of ABS shells needed to build the phantoms. Moreover, the results also confirm that the envisaged device, while quite simple, being made by a single line of antennas, is capable of achieving real-time 3D imaging results conveying information on the position of the ablated area. It is worth noting that this is, in any case, remarkable, as monitoring liver ablation has a number of additional difficulties compared to monitoring other MWA treatments considered in the literature [20,21,22] due to the impossibility of measuring the signal all around the ROI and the high level of losses in the interested tissue. Moreover, it is worth noting here that to mimic actual clinal conditions, the pre-treatment scenario should consider tumor tissue rather than liver tissue. However, as the first stage of validation of an ablation monitoring device typically involves ex-vivo liver samples (e.g., [12]), healthy liver tissue was chosen as a pre-treatment scenario in the simulation study.

Thanks to its simplicity, the proposed microwave imaging system shows advantages in terms of cost-effectiveness in comparison with conventional modalities like CT and MRI, whereas it is not affected by the hyper-echogenic cloud caused by ablation treatment that blinds US sensors. Moreover, the real-time imaging provided by the MWI system can be helpful for the clinician to control the ablation treatment. However, while US can image the whole ablated area thanks to the higher penetration depth, the achieved results suggest that the proposed device is mostly able to locate the ablated area boundary from the side of the antenna array. Accordingly, improvements in the image quality are needed to provide fully meaningful clinical information. To this end, a number of improvements can be foreseen. First of all, to improve resolution along the *y*-axis and the imaging of the non-illuminated side of the ablation region, the array elements can be displaced not along a line but with planar arrangement (for instance, placing the eight antennas on two parallel lines). Moreover, a larger number of frequency points can be acquired to increase the amount of data and improve rejection to noise. Improvements can also be foreseen for the imaging algorithm, either using a different algorithm, such as the iterated version of the DBA used in [22], or incorporating available a priori information in the electromagnetic model used to build the imaging kernel ***K***. In this respect, the use of a pre-operatory image of the scenario can be extremely useful.

## 5. Conclusions

This contribution analyses the pros and cons of a proposed arrangement for developing experiments with a multi-view multi-static MWI system for real-time monitoring of thermal ablation of liver tumors. Overall, the observed results confirm that the considered setup was a valid “playground” to test the performance of the designed device. The conducted analysis showed that several improvements can be implemented on the proposed setup, both from the point of view of the hardware of the system and of the data processing technique. Future work will address the realization of the experimental setup (i.e., the phantom), the coupling medium, and antennas, also considering different positioning of the antennas as derived from this study.

## Figures and Tables

**Table 1:** L2 norm of scattering matrices of the simulated data set.

Frequencies (MHz)	a1	a2	b1	b2	c1	c2	s0
**600**	0.6110	0.611	0.611	0.5958	0.6105	0.5956	0.6106
**800**	0.5935	0.5938	0.5935	0.5831	0.5929	0.5826	0.5932
**1000**	0.6027	0.6028	0.6026	0.5829	0.6017	0.5828	0.6010
**1200**	0.5581	0.5573	0.5581	0.5583	0.5581	0.5564	0.5550
**1400**	0.2689	0.2725	0.2685	0.2641	0.2718	0.269	0.2769

**Table 2:** L2 norm (in dB) of the differential scattering matrices of the simulated data sets aX-s0, bX-s0, and cX-s0, with X being equal to 1 or 2, normalized to the s0 data set. Refer to Figure 1 for the different cases.

Frequencies (MHz)	(a1-s0)/s0	(a2-s0)/s0	(b1-s0)/s0	(b2-s0)/s0	(c1-s0)/s0	(c2-s0)/s0
**600**	−26.74	−40.45	−27.12	−31.37	−30.37	−30.85
**800**	−31.60	−36.28	−31.77	−32.99	−33.88	−33.46
**1000**	−28.65	−30.87	−27.97	−28.01	−33.78	−29.56
**1200**	−26.79	−27.24	−25.94	−28.44	−29.97	−31.84
**1400**	−17.72	−21.17	−16.98	−14.04	−18.40	−16.06

**Table 3:** L2 norm (in dB) of the differential scattering matrices of the simulated data sets bX-aX and cX-bX, normalized to the s0 data set. Refer to Figure 1 for the different cases.

Frequencies (MHz)	(b1-a1)/s0	(b2-a2)/s0	(c1-b1)/s0	(c2-b2)/s0
**600**	−54.13	−31.26	−35.63	−40.75
**800**	−49.44	−32.00	−36.48	−32.67
**1000**	−46.63	−29.09	−34.21	−30.47
**1200**	−43.06	−27.35	−33.67	−30.54
**1400**	−35.78	−15.50	−24.87	−23.05

**Table 4:** NMSE with respect to the ideal reconstruction.

a1-s0	b1-s0	c1-s0	b1-a1	c1-a1
0.09	0.09	0.06	2.13	0.27
**a2-s0**	**b2-s0**	**c2-s0**	**b2-a2**	**c2-a2**
0.25	0.36	0.66	1.37	0.26
